# Factors Affecting the Risk of Diabetes Development among Brazilian Residents in Japan

**DOI:** 10.3390/ijerph19137698

**Published:** 2022-06-23

**Authors:** Satoko Mizohata, Yuko Uesugi, Hiroya Matsuo

**Affiliations:** 1 Department of Public Health, Graduate School of Health Sciences, Kobe University, Kobe 654-0142, Japan; uesugi@pearl.kobe-u.ac.jp; 2 Department of Nursing, Kinjo Gakuin University, Nagoya 463-8521, Japan; 3 Department of Nursing, Osaka Shin-Ai College, Osaka 538-0053, Japan; matsuoh@osaka-shinai.ac.jp

**Keywords:** diabetes, risk factor, Brazilian, risk score, *feijoa*

## Abstract

This study aimed to uncover the risk of diabetes and its associated factors among Brazilian residents in Japan. An anonymous questionnaire survey was conducted among Brazilians living in Japan who were aged >40 years. The questionnaire collected data on the participants’ Finnish Diabetes Risk Score (FINDRISC), basic demographics, and health conditions. The analysis was based on the FINDRISC to assess factors affecting the risk of diabetes. Among the 181 participants (mean age, 52.9 years), 77 and 104 were men and women, respectively; 26 (14.3%) people were diagnosed with diabetes. The participants were categorized into high- and low-risk groups based on a FINDRISC value of ≥12 and ≤11, respectively. The high-risk group, 26 [17.7%] patients, contained a significantly higher proportion whose education level was less than elementary school, who were unable to speak Japanese, and whose diets contained little *feijoa* than the low-risk group. *Feijoa* is a local Brazilian bean dish that is low in fat and calories but contains high protein content, which helps prevent diabetes. Our findings suggest that increased health literacy is important for controlling chronic diseases, including diabetes.

## 1. Introduction

The prevalence of diabetes is increasing worldwide with lifestyle changes and an aging population. In 2019 alone, nearly 463 million people were diagnosed with diabetes. The prevalence of diabetes among adults aged between 20 and 79 years is 9.3% [1], with estimates showing that approximately 700 million people will be diagnosed annually with diabetes by 2045 if no appropriate interventions for non-communicable diseases such as diabetes are developed [2]. Diabetes is a potent risk factor for the development of cardiovascular disease and is associated with impaired lipid metabolism, which leads to a further increase in cardiovascular disease risk [3,4].

Brazil is a country with the fifth largest number of people with diabetes after China, India, the United States, and Pakistan. Approximately 8.1% of the total population of Brazil has diabetes. According to a recent report by the World Health Organization (WHO), the prevalence of diabetes based on relevant risk factors is as follows: overweight (54.2%), obesity (20.1%), and physical inactivity (27.2%) [5]. Another study has estimated that approximately 16.8 million people currently have diabetes, and 7.7 million individuals remain undiagnosed in Brazil [6].

The number of Japanese individuals with diabetes has also been increasing; a total of 3,289,000 had been diagnosed with diabetes in 2017 [7]. Moreover, evidence suggests that 19.7% of all men and 10.8% of all women are strongly suspected of having diabetes, with the rate being higher in older adults [8]. An epidemiological study conducted on Japanese individuals has shown that the risk factors for diabetes development are as follows: aging, family history, obesity, physical inactivity, and impaired glucose tolerance [9].

The number of foreigners living in Japan has been increasing every year. Approximately 2.88 million foreign residents had been documented by the end of 2020; of them, approximately 200,000 (9.7%) were Brazilian [10]. Historically, many Japanese people have immigrated to Brazil and vice versa. Brazil was undergoing an economic crisis in the 1980s and 1990s; Japan’s Immigration Control Act was amended in 1990 to create the “permanent resident” status. Thus, many Brazilian immigrants who had come to Japan as workers in the 1980s and 1990s are now in their 50s and 60s. Most Brazilians are employed part-time or work night shifts, which tend to result in irregular lifestyles.

The diet of Japanese individuals living in Brazil (Japanese-Brazilians) has already been westernized, increasing the risk of cardiovascular diseases [11]. Diabetes increases the risk of death [12]. Japanese-Brazilians have a higher prevalence of diabetes and mortality than Japanese individuals living in Japan; the first- and second-generation individuals in the former group have higher aforementioned rates than age-matched individuals in the latter group [13]. However, to the best of our knowledge, no studies have assessed the risk of diabetes among Brazilians living in Japan.

Previous studies have shown that there are Brazilians living in Japan who suffer from lifestyle-related diseases [14,15]. Presently, most Brazilian residents are in their 40s and 50s, and it is predicted that diabetes will cause severe health issues among Brazilian residents in Japan in the near future, due to aging and the prevalence of lifestyle-related diseases. Evaluation of the risk of diabetes development may facilitate early detection and preventive interventions such as health education for Brazilian residents. Therefore, the present study sought to determine the factors associated with the risk of type 2 diabetes development among Brazilian residents in Japan.

## 2. Materials and Methods

### 2.1. Research Design

This study had a cross-sectional survey design.

### 2.2. Participants

Our participants included Brazilians living in Japan who were aged ≥40 years. The exclusion criteria were individuals with dementia or mental illness. Specific health checkups—health examinations focusing on metabolic syndromes to prevent lifestyle-related diseases—are performed for people aged ≥40 years in Japan. Given that diabetes is more prevalent among those in their 40s, people aged >40 years were included in this study.

### 2.3. Survey Method

Between September 2020 and January 2021, unmarked questionnaires were mailed to the participants and responses were requested using enclosed return envelopes. The participants were recruited via their Japanese-Brazilian acquaintances, nonprofit organizations (NPOs) that support Brazilians, and temporary employment agencies that employ Brazilians; the participants received explanations regarding the purpose of this study and the questionnaire to be distributed.

### 2.4. Instruments

The present study employed a self-administered questionnaire to collect relevant information.

#### 2.4.1. Finnish Diabetes Risk Score

In this study, the Finnish Diabetes Risk Score (FINDRISC) questionnaire, which was developed by Finnish researchers in 2003 [16], was used to evaluate the risk of diabetes among the participants. The FINDRISC questionnaire comprises eight items that determine and rate the risk of developing type 2 diabetes mellitus within 10 years [16]. These items include age; body mass index (BMI); abdominal circumference; daily physical activity; daily intake of vegetables, fruits, and berries; use of antihypertensive drugs; history of hyperglycemia; and family history of diabetes. The responses to each item were scored according to their influence on diabetes development. The total score ranges from 0 to 26 points, and risk groups are classified as follows: <7 points, low risk (1 in 100 people will develop disease); 7–11 points, slightly elevated risk (1 in 25 people will develop the disease); 12–14 points, moderate risk (1 in 6 people will develop the disease); 15–20 points, high risk (1 in 3 people will develop the disease); and >20 points, very high risk (1 in 2 people will develop the disease). This questionnaire has been used as a tool for evaluating diabetes risk; it is the most effective and inexpensive tool recommended by the International Diabetes Federation for identifying, without clinical examinations, individuals at high risk of diabetes development. This tool has been validated in Europe and the United States as well as in Finland, Spain, Belgium, Greece, India, Mexico, Brazil, and Japan to study the risk of diabetes development. This study used the original FINDRISC questionnaire, which was translated into Portuguese.

#### 2.4.2. Attributes

The questionnaire was prepared based on existing documents [17], previous studies [18,19,20,21], and the National Nutrition Questionnaire [8], which was developed by the Ministry of Health, Labor and Welfare in Japan. The items include basic attributes such as age, sex, family, educational background, length of stay in Japan, and Japanese communication skills; health status such as medical history and health consciousness; lifestyle such as work, exercise, smoking, and diet; BMI; and abdominal circumference.

#### 2.4.3. Data Analysis

The participants were categorized into high- and low-risk groups based on a FINDRISC of ≥12 and ≤11, respectively. A score of ≥12 indicates moderate risk, and the cut-off risk score of 11 can be used to identify undiagnosed diabetes with a sensitivity of 66% in men and 70% in women [22]. Another study has suggested that FINDRISC cut-off values of ≥12 have a sensitivity and specificity of 100% and 84.1%, respectively, for detecting glycemic abnormalities [23]. The confidence level of the significance test was set at 95% (*p* < 0.05). The software SPSS version 27 for Windows (IBM, Armonk, NY, USA) was used for all the statistical analyses.

### 2.5. Text Translation

The questionnaire was translated to Portuguese by a Japanese-Brazilian who is fluent in Japanese and has lived in Japan for 8 years, followed by confirmation from another Japanese-Brazilian who has lived in Japan for 15 years and works in the educational sector.

### 2.6. Ethical Consideration

A research cooperation request form describing the purpose, significance, and ethical considerations of this study was enclosed with the questionnaire, and consent was determined based on the participants’ decision to complete and return the questionnaire. All these aforementioned documents were written in Portuguese. This study was approved by the Institutional Review Board of Kobe University (approval number, 896).

## 3. Results

The questionnaire was distributed to a total of 477 participants; of them, 227 responded (response rate, 47.5%). After excluding incomplete questionnaires, 181 participants were finally investigated (valid rate, 79.7%), including 77 men and 104 women (age: mean ± standard deviation [SD], 52.9 ± 8.4 years).

### 3.1. Basic Demographics of the Participants

The demographics of the participants are summarized in Table 1. Among the included participants, 77 and 104 were men and women, respectively, with a mean age of 52.9 (SD = 8.4) years. Moreover, 70 (38.7%), 77 (42.5%), 25 (13.8%), and 9 (5.0%) participants were in their 40s, 50s, and 60s and aged ≥70 years, respectively. The highest number of Japanese descendants were those in their second generation (99 [54.7%]), followed by those in their third generation (53 [29.3%]), which together accounted for approximately 80% of the total population.

The average length of stay in Japan was 23.4 years, with the most frequently reported length of stay being 20–29 years for 86 (47.5%) participants. Moreover, 45 (24.9%) participants had been in Japan for >30 years.

In terms of educational background, the largest proportion of participants comprised those who had graduated from high school (65 [35.9%]), followed by those who had graduated from college or university (63 [34.8%]), vocational school (20 [11.0%]), junior high school (19 [10.5%]), and elementary school (13 [7.2%]). In terms of the level of Japanese language proficiency, 63 (34.8%) participants could speak Japanese fluently. Approximately 70% of the participants, including 63 (34.8%) who were fluent in conversational Japanese, could conduct daily conversations, whereas approximately 90%, including 46 (25.4%) who engaged in simple daily conversations, had mastered conversational Japanese. A total of 10 (5.5%) participants could not understand Japanese at all. In terms of Japanese reading comprehension, 33 (18.2%), 49 (27.1%), 36 (19.9%), and 18 (8.3%) participants, respectively, could read newspapers, manga and picture books, product names, or could not read at all.

Most of the participants (82 [45.5%]) earned between 2–4 million yen, and approximately 60% of them earned <4 million yen, including 24 (13.3%) who earned <2 million yen. Moreover, 35 (19.3%), 3 (1.7%), and 4 (2.2%) participants had incomes of 4–6, 6–8, and >10 million yen, respectively.

### 3.2. Health Conditions and Lifestyle-Related Disease

Among the 181 participants, 56 (30.9%) had some type of disease; of them, 26 (14.4%) (10 men and 16 women) had been diagnosed with diabetes. Among them, 14 (7.7%) were treated with insulin.

After evaluating their own health condition on a 5-point Likert scale, approximately 70% (69.6%) of the participants reported that their health was “very good” or “good.” When asked what they thought of their own body shape, 48.1%, 39.2%, and 4.4% participants reported that they were normal, slightly overweight, and overweight, respectively.

Accordingly, 3 (1.8%), 76 (45.5%), 65 (38.9%), and 23 (13.8%) participants had a BMI of <18.5, 18.5–25, 25–30, and >30 kg/m^2^, respectively. The mean BMI of the participants was 25.5 kg/m^2^. In terms of BMI and self-perception, all three participants with a BMI of <18.5 kg/m^2^ considered themselves “normal.” Of the 76 participants with a BMI of 18.5–25 kg/m^2^, >50% perceived themselves as “normal.” However, 13 participants perceived themselves as “too thin” or “slightly too thin.” Notably, nine participants perceived themselves as “slightly overweight.” Among the 65 participants with a BMI between 25 and <30 kg/m^2^, 23 perceived themselves as “normal” and 42 as “slightly overweight.” Meanwhile, among the 23 participants with a BMI of >30 kg/m^2^, two perceived themselves as “normal,” 12 as “slightly overweight,” and eight as “overweight.”

The mean abdominal circumference was 90.3 cm (SD = 12.9) and 81.3 cm (SD = 12.3) for men and women, respectively.

Table 2 presents details on the lifestyle of the participants. A total of 93 (51.4%) of the 118 participants had an exercise habit. The most common durations of sedentary time per day were <3 h for 74 (40.9%), 3–8 h for 54 (29.8%), and <8 h for 18 (9.9%) participants. The most common durations of time spent walking or standing in a day were >8 h for 103 (56.9%), <3 h for 22 (12.24%), and 3–8 h for 37 (20.4%) participants.

Of the 181 participants, nearly 80% of the 138 participants (76.2%) consumed breakfast daily, whereas approximately 10% (17 [9.4%]) did not eat breakfast. Moreover, 44 (24.3%) participants consumed snacks every day, whereas 44 (24.3%) did not. Weekday meal times were regular for 123 (68.0%) and irregular for 8 (4.4%) respondents. The frequency of Japanese food consumption in a week was 1–4 days per week for 78 respondents (43.3%) (the largest group), ≥5 days for 61 (33.7%), and rarely for 41 (22.7%). Moreover, the frequency of *feijoa* consumption, which is commonly consumed in Brazil, was as follows: 80 (44.2%) consumed it rarely, 66 (36.5%) consumed it 1–4 days per week, and 23 (12.7%) consumed it ≥5 days per week. Regarding the seasoning of rice, as is common in Japan, the highest number of participants (151 [83.4%]) did not add any seasoning; 10 (5.5%) participants used only salt; 3 (1.7%) used salt and oil; 1 (0.6%) used salt and garlic; and 16 (8.8%) used salt, oil, and garlic.

### 3.3. FINDRISC

The participants’ FINDRISCs based on their age are shown in Table 3. The scores were as follows: 58 (32.0%) scored ≤7, 65 (35.9%) scored 7–11, 28 (15.5%) scored 12–14, 20 (11.0%) scored 15–19, and 10 (5.5%) scored ≥20. After excluding 26 people who were already diagnosed with diabetes, the low- and high-risk groups comprised 123 (68.0%) and 32 participants (17.7%), respectively.

### 3.4. Comparison of Various Factors between High- and Low-Risk Groups Based on FINDRISC

As mentioned earlier, the participants were divided into two groups according to their FINDRISCs: a low-risk group (≤11) and a high-risk group (≥12). Subsequently, differences in the proportion of participants in the two groups were evaluated in terms of their length of stay in Japan, education, Japanese language ability, employment status, and income (Table 4).

Regarding educational background, a significant difference was observed between the groups in the proportions of participants who received only elementary school education and those who received more than primary school education (*p* = 0004). Regarding the ability to speak Japanese, a significant difference was observed in the proportions of participants who spoke and those who did not speak Japanese (*p* = 010). No significant differences were noted between the groups in terms of the length of stay, Japanese language ability (reading), employment status, or income.

Regarding factors associated with eating habits, no significant differences were noted between the groups in terms of the consumption of breakfast and snacks, regularity of meal intake, frequency of Japanese food consumption, or seasoning of rice. However, a significantly high number of people in the low-risk group consumed *feijoa* >5 days per week (*p* = 0.035).

## 4. Discussion

To the best of our knowledge, this study is the first to assess factors associated with an increased risk of diabetes development among Brazilian residents in Japan.

### 4.1. Health Issues and Lifestyle-Related Diseases

The proportion of any disease among our participants was 30%; the most common diseases were hypertension, cardiovascular disease, and diabetes. A previous study on Brazilian residents in Japan reported that 50% of the participants had any disease such as hypertension, urinary tract stones, and cardiovascular disease [24]. Most of the diseases were related to the participants’ lifestyle, with the prevalence of diabetes being particularly high (i.e., 13% in men and 15.4% in women). The prevalence of diabetes among the Japanese is 18.7% in men and 9.3% in women [8]. However, the prevalence of diabetes in Brazil has been reported to be 8.1% [5]). This suggests that the prevalence of diabetes is high among Brazilian residents in Japan.

Considering changes in lifestyle, including diet and exercise patterns, in the population, type 2 diabetes is no longer a disease developing in the middle or older age; it has been increasingly diagnosed in young individuals [25]. Recent studies have shown that compared with older age, a younger age at diabetes diagnosis is associated with a higher risk of mortality and vascular disease development. Therefore, in the future, surveys must be conducted among young individuals.

In terms of self-reported health cognition, 70% of the study participants responded that their health was good. However, some difference was noted between their body image and BMI. The mean BMI of the participants was 25.5 kg/m^2^. Among the 88 participants (52.7%) with a BMI of ≥25 kg/m^2^, 25 (15%) reported that they had an “appropriate weight.” A previous study has reported that men and women in Japan have mean BMIs of 23.8 and 25.5 kg/m^2^, respectively [26]. Given that the genetic composition of Japanese-Brazilians was similar to that of the Japanese, obesity factors were supposedly associated with lifestyle-related activities.

Nearly 50% of the participants exercised regularly and frequently, with their exercise activities being higher than the mean exercise data for Japanese individuals (33.4% and 25.1% in men and women, respectively) [8]. Moreover, our participants had less sedentary time (the duration >3 h for 40% participants), and 60% had a walking and standing time of >8 h. Given that most of the Brazilians living in Japan are working in the production sector or as laborers [27], their activity levels can be expected to be high.

Approximately 80% of the participants consumed breakfast every day, and nearly 70% had a set mealtime on weekdays. This routine is likely because of the work-centered lifestyle they take for granted and the regularity of their daily routine. Japanese food was consumed by approximately 70% of the participants. However, approximately 50% had a habit of consuming *feijoa*, which is a bean dish eaten commonly in Brazil. This fact indicates that their eating habits are a mixture of Japanese and Brazilian habits, considering that they have been residing in Japan for an average of 25 years. Rice is also customarily consumed in Brazil, although the Brazilian style involves cooking the rice with salt, oil, and garlic. In this survey, approximately 80% of the participants answered that they do not season their food, which shows that they are influenced by Japanese eating habits.

### 4.2. Factors Associated with an Increased Risk of Diabetes Development

Among the 181 participants included in the study, 17.7% were in the high-risk group. A 2018 survey study conducted in Brazil using FINDRISC [28] reported that 22.7% of the participants belonged to high-risk groups. These results indicate that the risk of diabetes is also not low among Brazilians living in Japan. This result was possibly because many of the participants are work-based immigrants and lead relatively regular lives and that many of them have Japanese eating habits.

Significant differences were observed between the high- and low-risk groups in terms of education and Japanese language ability (speaking), suggesting that lower education and Japanese language ability were associated with a higher risk of diabetes development. The lack of education may predispose individuals to a higher risk of diabetes because of a poor understanding of the disease and its prevention, which in turn leads to poorer health behaviors. Indeed, low health literacy has been associated with poorer health outcomes and poorer use of healthcare services [29].

In terms of Japanese language ability, Japanese reading comprehension of the participants was lower than their speaking ability. For Japanese-Brazilians who are usually conversational, this might not have been a factor for increased diabetes risk given that they receive less information in writing than through conversation. However, a significant difference in diabetes risk was observed between those who could speak conversational Japanese and those who could not speak Japanese at all. Our findings showed that those who could not speak Japanese at all were at a higher risk of diabetes than those who could, given that the latter group was likely to receive more information through conversation. The WHO has reported that foreign residents are more likely to be marginalized in their host societies and are less likely to benefit from health services [30]. Therefore, people who cannot converse at all might not be receiving sufficient information on diabetes and its preventive measures.

### 4.3. Eating Habits

No significant differences in the consumption of breakfast and snacks, regularity of meal intake, frequency of Japanese food consumption, or seasoning of rice was observed. However, those who consumed *feijoa* ≥5 days a week had a significantly lower risk for diabetes development than those who did not. This is perhaps because *feijoa*, a bean dish, is low in fat and calories but is rich in protein, which helps reduce the risk of diabetes and cardiovascular disease.

### 4.4. Limitations

Given the difficulty in contacting all Brazilians living in Japan, we targeted Japanese areas with many Brazilians. However, owing to the coronavirus disease 2019 (COVID-19), we could only approach a limited number of individuals, which might not be representative of the target population. We had originally planned to distribute the questionnaires in person to measure height, weight, and abdominal circumference; however, we had to obtain data via self-reported questionnaires because of COVID-19-related restrictions. Given that diabetes is most common in people in their 40s, this study included people aged >40 years. Nonetheless, recent evidence suggests that this disease has been frequently diagnosed in younger age groups; therefore, we might have missed a part of our statistical population, which might have affected our results. Future studies should include younger age groups. Nevertheless, the data obtained in this study are valuable because, to the best of our knowledge, the risk of diabetes in Brazilian residents in Japan has not been studied previously. Because we could not obtain sufficient data on the dietary habits of this population, future studies should include this variable.

### 4.5. Suggestions

Basic and health education is crucial. Thus, health literacy is pivotal for improved knowledge and understanding of diabetes and other health-related issues, which facilitates disease prevention disease and health maintenance.

## 5. Conclusions

To the best of our knowledge, this study is the first to determine factors associated with an increased risk of diabetes development among Brazilian residents in Japan. The risk factors included less than elementary school education, poor ability in conversational Japanese, and little or no consumption of *feijoa*. Information regarding diabetes is crucial for its prevention. Our findings suggest that increased health literacy is essential for controlling chronic diseases.

## Figures and Tables

**Table 1:** Participant demographics.

Characteristics	*n* (%)
Sex	
Male	77 (42.5)
Female	104 (57.5)
Age (years), mean ± SD	52.9 ± 8.4
40s	70 (38.7)
50s	77 (42.5)
60s	25 (13.8)
>70	9 (5.0)
Japanese descent	
1st (1sei)	5 (2.8)
2nd (2sei)	99 (54.7)
3rd (3sei)	53 (29.3)
4th (4sei)	2 (1.1)
Non-Japanese	22 (12.2)
Period of stay in Japan (years; mean, 23.4)	
<5	17 (9.4)
5–9	2 (1.1)
10–19	29 (16.0)
20–29	86 (47.5)
>30 years	45 (24.9)
Unanswered	2 (1.1)
Educational background	
Elementary school	13 (7.2)
Junior high school	19 (10.5)
High school	65 (35.9)
Vocational school	20 (11.0)
University or above	63 (34.8)
Unanswered	1 (0.6)
Japanese proficiency	
(Conversational)	
Fluent	63 (34.8)
Daily conversation	62 (34.3)
Simple daily conversation	46 (25.4)
Incomprehensible	10 (5.5)
(Reading comprehension)	
Ability to read newspapers	33 (18.2)
Ability to read manga and picture books	49 (27.1)
Ability to read product names	48 (26.5)
Incomprehensible	36 (19.9)
Unanswered	15 (8.3)
Household annual income (million yen)	
<2	24 (13.3)
2–4	82 (45.3)
>4–6	35 (19.3)
>6–8	15 (8.3)
>8–10	3 (1.7)
>10	4 (2.2)
Unanswered	18 (9.9)

**Table 2:** Lifestyle habits of the study participants.

Lifestyle Habits	*n* (%), N = 181
Exercise habit	
Yes	93 (51.4)
Sedentary time (hours per day)	
<3	74 (40.9)
3–8	54 (29.8)
>8	18 (9.9)
Unanswered	35 (19.3)
Walking and standing time (hours per day)	
<3	22 (12.2)
3–8	37 (20.4)
>8	103 (56.9)
Unanswered	19 (10.5)
Breakfast	
No breakfast	17 (9.4)
Occasional breakfast	26 (14.4)
Breakfast every day	138 (76.2)
Snacking	
No snacking	44 (24.3)
Occasional snacking	93 (51.4)
Snacking every day	44 (24.3)
Weekday mealtimes	
Regular	123 (68.0)
Occasionally irregular	50 (27.6)
Irregular	8 (4.4)
Japanese food consumption (days per week)	
≥5	61 (33.7)
1–4	78 (43.1)
Rare	41 (22.7)
*Feijoa* consumption (days per week)	
≥5	23 (12.7)
1–4	66 (36.5)
Rare	80 (44.2)
Rice seasoning	
No seasonings	151 (83.4)
Salt	10 (5.5)
Salt + oil	3 (1.7)
Salt + garlic	1 (0.6)
Salt + oil + garlic	16 (8.8)

**Table 3:** FINDRISC distribution stratified based on age.

		Risk Scores					
		<7	7–11	12–14	15–19	19<	Total
Age (years)	40–49	21	27	14	7	1	70
	50–59	32	28	7	7	3	77
	60–69	5	10	4	4	2	25
	>70	0	0	3	2	4	9
Total		58	65	28	20	10	181

≤7, low risk; 7–11, slightly elevated risk; 12–14, moderate risk; 15–20, high risk; and >20, very high risk.

**Table 4:** Comparison of various factors between the high- and low-risk groups based on FINDRISCs.

Factors		Low-Risk Group	High-Risk Group	*p*-Value
		*n* (%)	*n* (%)	
Length of stay (years)	>5	11 (21.64)	4 (7.8)	0.083
	<30	33 (64.7)	3 (5.9)	
Educational background	Only primary school	2 (1.3)	5 (3.2)	0.004
	Secondary school or above	121 (78.1)	27 (17.4)	
Japanese speaking ability	Can speak	120 (77.4)	27 (17.4)	0.010
	Unable to speak	3 (1.9)	5 (3.2)	
Japanese reading ability	Can read	90 (63.4)	22 (15.5)	0.656
	Unable to read	23 (16.2)	7 (4.7)	
Employment type	Full-time	99 (70.2)	21 (14.9)	0.093
	Less than full-time	14 (9.9)	7 (5.0)	
Income (million yen)	≤2	15 (10.4)	5 (3.5)	0.499
	>2	101 (70.1)	23 (16.0)	
Breakfast	Every day	95 (61.3)	24 (15.5)	0.790
	Occasional/no breakfast	28 (18.1)	8 (5.2)	
Snacking	Every day	33 (21.3)	5 (3.2)	0.139
	Occasional/no snacking	90 (58.1)	27 (17.4)	
Dietary regularity	Regular	88 (56.8)	19 (12.3)	0.185
	Occasionally irregular/irregular	35 (22.6)	13 (8.4)	
Japanese food consumption (days per week)	≥5	44 (28.6)	7 (4.5)	0.163
	≤4	79 (51.3)	24 (15.6)	
Rice seasoning	Yes	23 (14.8)	5 (3.2)	0.801
	No	100 (64.5)	27 (17.4)	
*Feijoa* consumption	≥5 days per week	19 (21.6)	1 (1.1)	0.035
	Rare	49 (55.7)	19 (21.6)	

## Data Availability

Not applicable.

## References

[1] IDF (International Diabetes Federation) (2019). IDF DIABETES ATLAS.

[2] WHO (World Health Organization) Global Report on Diabetes. https://www.who.int/publications/i/item/9789241565257.

[3] Jakubiak G.K., Cieślar G., Stanek A. (2022). Nitrotyrosine nitrated lipoproteins, and cardiovascular dysfunction in patients with type 2 diabetes: What do we know and what remains to be explained?. Antioxidants.

[4] Petrie J.R., Guzik T.J., Touyz R.M. (2018). Diabetes, hypertension, and cardiovascular disease: Clinical insights and vascular mechanisms. Can. J. Cardiol..

[5] WHO (2016). Global Diabetes Report: Country Profiles Brazil. https://www.who.int/publications/m/item/diabetes-bra-country-profile-brazil-2016.

[6] IDF Diabetes Atlas https://www.diabetesatlas.org/en/.

[7] Ministry of Health and Welfare Patient Survey. https://www.mhlw.go.jp/toukei/saikin/hw/kanja/17/index.html.

[8] Ministry of Health and Welfare National Health and Nutrition Examination Survey. https://www.mhlw.go.jp/stf/seisakunitsuite/bunya/kenkou_iryou/kenkou/eiyou/r1-houkoku_00002.html.

[9] Ministry of Health and Welfare Long-Term Chronic Diseases Research Project Diabetes Survey. Research Report. https://www.mhlw.go.jp/www1/topics/kenko21_11/b7.html.

[10] Ministry of Justice, Japan https://www.moj.go.jp/isa/policies/statistics/toukei_ichiran_touroku.html.

[11] Mizushima S., Moriguchi E.H., Ishikawa P., Hekman P., Nara Y., Mimura G., Moriguchi Y., Yamori Y. (1997). Fish intake and cardiovascular risk among middle-aged Japanese in Japan and Brazil. J. Cardiovasc. Risk.

[12] Suely G.A., Osiro K., Matsumura L., Massimino F.C., Ferreira S.R.G. (2005). Japanese-Brazilians diabetes study group. Glucose intolerance and all-cause mortality in Japanese migrants. Diabetes Res. Clin. Pract..

[13] Ferreira S.R., Iunes M., Franco L.J., Iochida L.C., Hirai A., Vivolo M.A. (1996). Disturbances of glucose and lipid metabolism in first and second generation Japanese-Brazilians. Japanese-Brazilian diabetes study group. Diabetes Res. Clin. Prac..

[14] Marie T., Yoshie K. (2005). Health Status and Lifestyle of Foreign Attendants at the Medical Check-up for Foreigners in Nagano, 2003. Bull. Nagano Coll. Nurs..

[15] Asayama M., Suzuki S., Tsujioka Y., Kawai Y., Hatashita H. (2007). Medical Consultation Behavior and Lifestyle of Brazilians Living in Japan Who Visited Medical Institutions in Shiga Prefecture. Bull. Kohka Public Hosp..

[16] Lindström J., Tuomilehto J. (2003). The diabetes risk score: A practical tool to predict type 2 diabetes risk. Diabetes Care.

[17] Ministry of Health, Labor and Welfare https://www.mhlw.go.jp/stf/seisakunitsuite/bunya/kenkou_iryou/kenkou/seikatsu/seikatusyuukan.html.

[18] Shibazaki A., Mizuguchi M., Tagaya A. (2007). Lifestyle of Brazilian inhabitants in a rural area of Nagano prefecture with special reference to dietary habits [bulletin]. Nagano Coll. Nurs..

[19] De Almeida-Pititto B., Dias M.L., de Moraes A.C., Ferreira S.R., Franco D.R., Eliaschewitz F.G. (2015). Type 2 diabetes in Brazil: Epidemiology and management. Diabetes Metab. Syndr. Obes..

[20] Zhang Y., Pan X.F., Chen J., Xia L., Cao A., Zhang Y., Wang J., Li H., Yang K., Guo K. (2020). Combined lifestyle factors and risk of incident type 2 diabetes and prognosis among individuals with type 2 diabetes: A systematic review and meta-analysis of prospective cohort studies. Diabetologia.

[21] Sebastião E., Galvez P.A.E., Nakamura P.M., Papini C.B., Kokubun E., Gobbi S. (2017). Activity behavior, nutritional status and perceived health in older Brazilian adults: Does the number of chronic diseases matter?. Geriatr. Gerontol. Int..

[22] Saaristo T., Peltonen M., Lindström J., Saarikoski L., Sundvall J., Eriksson J.G., Tuomilehto J. (2005). Cross-sectional evaluation of the Finnish Diabetes Risk Score: A tool to identify undetected type 2 diabetes, abnormal glucose tolerance and metabolic syndrome. Diab. Vasc. Dis. Res..

[23] Vandersmissen G.J., Godderis L. (2015). Evaluation of the Finnish Diabetes Risk Score (FINDRISC) for diabetes screening in occupational health care. Int. J. Occup. Med. Environ. Health.

[24] Fukushima T., Moriyama M. (2003). Lifestyle-related health problems confronting Japanese-Brazilians immigrated to Japan. J. Jpn. Assoc. Rural Med..

[25] Nanayakkara N., Curtis A.J., Heritier S., Gadowski A.M., Pavkov M.E., Kenealy T., Owens D.R., Thomas R.L., Song S., Wong J. (2021). Impact of age at type 2 diabetes mellitus diagnosis on mortality and vascular complications: Systematic review and meta-analyses. Diabetologia..

[26] Ministry of Health and Welfare National Health and Nutrition Examination Survey. https://www.mhlw.go.jp/stf/houdou/0000177189_00001.html.

[27] Ministry of Internal Affairs and Communications Japan Number of Employees by Occupation. https://www.stat.go.jp/data/kokusei/2005/gaikoku/00/07.html.

[28] Correr C.J., Coura-Vital W., Frade J.C.Q.P., Nascimento R.C., Nascimento L.G., Pinheiro E.B., Ferreira W.M., Reis J.S., Melo K.F., Pontarolo R. (2020). Prevalence of people at risk of developing type 2 diabetes mellitus and the involvement of community pharmacies in a national screening campaign: A pioneer action in Brazil. Diabetol. Metab. Syndr..

[29] Berkman N.D., Sheridan S.L., Donahue K.E., Halpern D.J., Crotty K. (2011). Low health literacy and health outcomes: An updated systematic review. Ann. Intern. Med..

[30] World Health Organization (WHO) International Migration. https://apps.who.int/iris/bitstream/handle/10665/42793/9241562536.pdf?sequence=1.

